# n-Player Stochastic Duel Game Model with Applied Deep Learning and Its Modern Implications

**DOI:** 10.3390/s22062422

**Published:** 2022-03-21

**Authors:** Manik Gupta, Bhisham Sharma, Akarsh Tripathi, Shashank Singh, Abhishek Bhola, Rajani Singh, Ashutosh Dhar Dwivedi

**Affiliations:** 1 Chitkara University School of Engineering & Technology, Chitkara University, Himachal Pradesh, India; manik.gupta@chitkarauniversity.edu.in (M.G.); akarsh1278.be20@chitkarauniversity.edu.in (A.T.); 2 Department of Computer and Communication Engineering, Manipal University Jaipur, Jaipur, Rajasthan, India; shashank.singh@jaipur.manipal.edu; 3 Department of Computer Science and Engineering, Koneru Lakshmaiah Education Foundation, Vaddeswaram, Andhra Pradesh, India; abhishek_bhola@kluniversity.in; 4 Centre for Business Data Analytics, Department of Digitalization, Copenhagen Business School, 2000 Frederiksberg, Denmark; rs.digi@cbs.dk

**Keywords:** stochastic duel, video games, combat analysis, deep learning, trust calculation, game theory, LSTM

## Abstract

This paper provides a conceptual foundation for stochastic duels and contains a further study of the game models based on the theory of stochastic duels. Some other combat assessment techniques are looked upon briefly; a modern outlook on the applications of the theory through video games is provided; and the possibility of usage of data generated by popular shooter-type video games is discussed. Impactful works to date are carefully chosen; a timeline of the developments in the theory of stochastic duels is provided; and a brief literature review for the same is conducted, enabling readers to have a broad outlook at the theory of stochastic duels. A new evaluation model is introduced in order to match realistic scenarios. Improvements are suggested and, additionally, a trust mechanism is introduced to identify the intent of a player in order to make the model a better fit for realistic modern problems. The concept of teaming of players is also considered in the proposed mode. A deep-learning model is developed and trained on data generated by video games to support the results of the proposed model. The proposed model is compared to previously published models in a brief comparison study. Contrary to the conventional stochastic duel game combat model, this new proposed model deals with pair-wise duels throughout the game duration. This model is explained in detail, and practical applications of it in the context of the real world are also discussed. The approach toward solving modern-day problems through the use of game theory is presented in this paper, and hence, this paper acts as a foundation for researchers looking forward to an innovation with game theory.

## 1. Introduction

**Figure 1:**
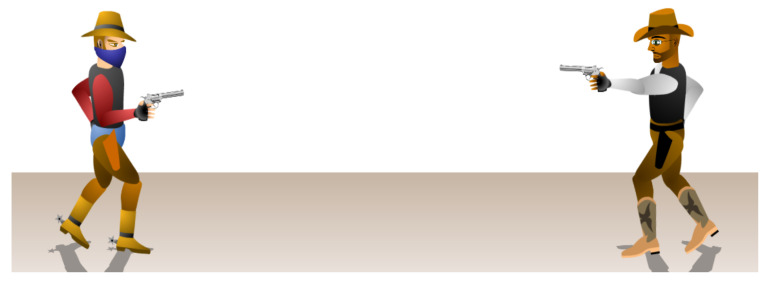
Stochastic duel setup.

Originally inspired by the works of Lanchester [1] and designed to analyze military operations and combat, the theory of stochastic duels [2] has come a long way. Various stochastic duel models have been introduced over the course of time based on the principles of the theory of stochastic duels, as shown in Table 1. Constrained environment variables, such as time and ammunition, were among the first variations of these models. Since the establishment of the fundamental stochastic duel game model [3], a variety of mathematical game models based on this theory have been developed to fulfill various applications. Figure 1 gives an idea of the environment of the fundamental stochastic duel in which the duelists can be seen pointing their weapons at each other. The fundamental stochastic duel game model describes a classic duel scenario where two players begin simultaneously with unloaded weapons and have unlimited access to ammunition and no time limits to score a kill.

### 1.1. Background

As depicted in Figure 1, a stochastic duel is a conflict between two players who fire at each other with fixed kill probability until one or both of them are dead.

Previously, stochastic duels were thought to be merely another way of analyzing simple and theoretical issues involving two-person combats or duels. It was essentially a combat evaluation tool that was supposed to either analyze the damage inflicted and received in combat or prescribe a hypothetical line of action for a military confrontation. As indicated in Table 1, there are several mathematical models based on the idea of stochastic duels that attempt to quantify the results of a duel in various ways. Other than stochastic duel theory, a few other attempts to analyze other forms of battle have been published, most of which are related to military operations and combat.

- Battle Damage Assessment: It is used to calculate the amount of damage inflicted by a fight, conflict or a bomb. After World War 1, battle damage assessment (BDA) became a need and a highly useful piece of information for all parties involved in the battle, and great effort was put into making it efficient [4]. Most of the work regarding BDA that is published in the public domain deals with aerial combat and bombings on a specific area through air, since an air strike causes major and widespread damage in a battlefield. The work conducted on this evaluation is still mostly focused on practical applications in war and conflict. As a result, it is not particularly dynamic in nature, and hence, it does not appear to have a wide range of use in other areas of battle analysis. It does, however, have a wide range of uses in terms of battles and bombs. Many major military technologies supplying companies base their early-warning defense systems on sensors that run on the principles of battle damage assessment.
- Munitions Effects Assessment: It refers to the effect of artillery and weapons on the enemy. This is another important tool for analyzing the damage inflicted by various weapon systems. However, it has a very limited use. Most of the work and models based on this assessment are either not available in the public domain, i.e., are not declassified by the military, or have a constrained usage mostly in specific weapon systems. Designing models for so many various types of weapon systems is unfeasible in the military technology business, and it presumably has no practical applications other than verifying the efficacy of newly designed weapon systems. However, this implies that the models based on the principles put forward by this assessment would prove extremely beneficial to the military for evaluating different weapon systems.
- Re-attack Recommendation: These models are used to predict the feasibility of a counterattack. In any conflict or duel, the defender has the option of launching a counterattack after successfully defending against an attack. These models usually incorporate a damage assessment, which is the amount of damage suffered when defending against an assault, as well as information about the attacker. Based on these parameters, such models calculate the probability of launching a successful counterattack. Since most of these models are bound to incorporate data and tactics sensitive to the state, information about them is scarce, and little work has been published in the public domain. It can be clearly observed that among the different models and evaluation techniques, the theory of stochastic duels is by far the most dynamic metric of them all.

### 1.2. Motivation of the Research

We formulate a model, which enables the theory of stochastic duels to have modern and civil applications. In the past, the civil and technological applications of models based on this theory seemed to be of a lower priority as compared to the military applications. Moreover, the idea of viewing civil and technological problems as duels is new and has not been worked upon very much yet. Most of the work on models based on this idea was conducted before the year 2000, and the world has seen some significant technological breakthroughs since then. As a result, a dynamic model is required to meet the demands of the modern world, while also leveraging the additional resources that are being added, as stated in Section 1.3.

### 1.3. Significance of Research in the Modern World

Aside from the military aspect, stochastic duels have also found their way into video games, since many genres tend to replicate the adrenaline rush caused in a battlefield. The principles put forward by the theory of stochastic duels and other military operations in general thrive in role-playing games (RPGs) and nearly the entire action-shooter genre of video games. Major video games in the “shooter” genre have adapted the concept of duels, which makes it possible to analyze these games and test the outcomes of different mathematical models. One of the major and early video game series that follow the concept of duels in the “shooter” genre is Counter Strike [5]. Since the original build, Counter Strike has seen an exponential growth in terms of the audience that it serves. Even the major modern games, such as Valorant and Player Unknowns’ Battlegrounds (PUBG) [6], follow similar principles.

The objectives of these video games tend to sound quite similar, but each game introduces its own unique selling point, which constitutes the introduction of novel parameters. CounterStrike, for example, is about a fight between organized and disorganized forces, such as counter-terrorists and terrorists. The goal of a counter-strike match is generally to eliminate the other team. During the elimination process, participants compete in duels for the duration of the game. As previously stated, the theory of stochastic duels may be used to examine these duels. This situation is very similar to that described in “A kind of stochastic duel model for guerrilla war” [7]. On the other hand, rather modern video games, such as PUBG, have a more complicated objective. In order to win a match of PUBG, a player is required to collect resources, engage and win in duels with other players and be the last player standing. Although there are many more aspects that go into determining a player’s chances of winning a PUBG match, the essential principle stays the same and can be examined using the theory.

**Figure 2:**
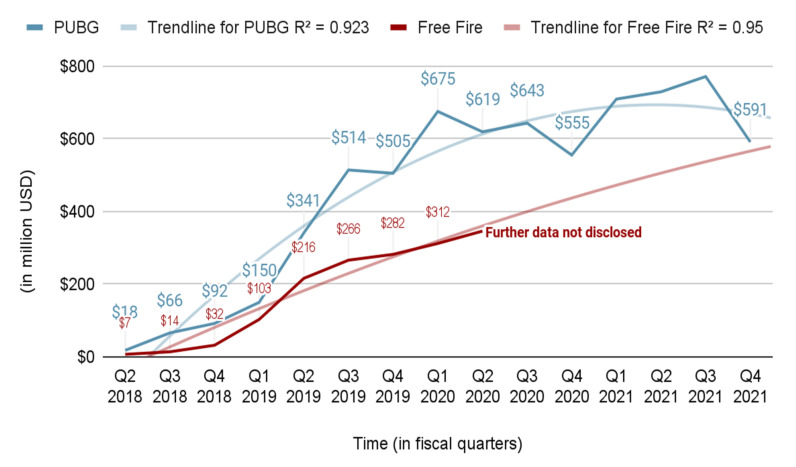
Growth in shooter games revenue (in USD million) [9,10].

Since the last decade (2010–2020), the gaming industry has shown an incredible up-run in terms of revenue, as well as interest among teenagers, young adults and adults. Over the past few years, and especially during the COVID-19 crisis, the gaming industry has seen rapid growth. Reports [8,9] suggest 400 million new users by 2023. Since most of these video games are privately held by different game studios, the revenue data are generally kept private, and hence, are hard to come by. Figure 2 shows the growth in revenue generated by different video games in the shooter genre based on publicly available data [10]. Trend lines for the plots are drawn with R-squared values close to 1, suggesting a reliable growth trend in the generated revenues. This is a telltale sign of increasing user engagement and interest.

**Figure 3:**
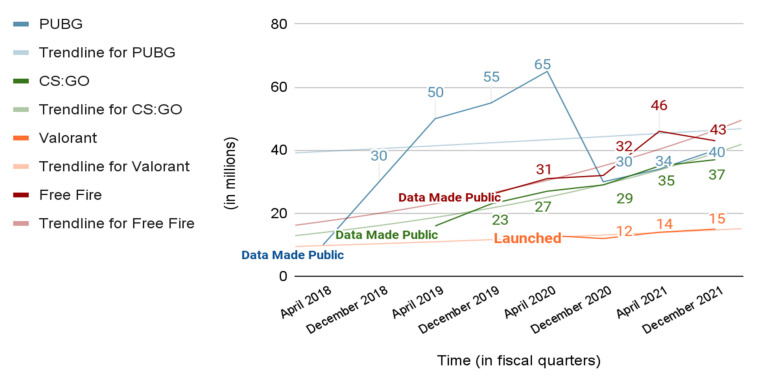
Growth in daily active users of different shooter-type video games.

As put forward by Tencent [11], the parent company of PUBG, the daily user activity has increased over the past years. Figure 3, generated using the data from Ref. [12], shows the growth in daily active users of different shooter-type video games [13,14]. It can clearly be observed that the game is gaining an audience from the trend lines in Figure 3, which suggest the potential for an exponential growth in daily active players. However, a significant drop in the daily active users of PUBG is evident in December 2020. This is explained by India’s ban on PUBG in September 2020, as it resulted in a major user base being taken away from the game.

**Figure 4:**
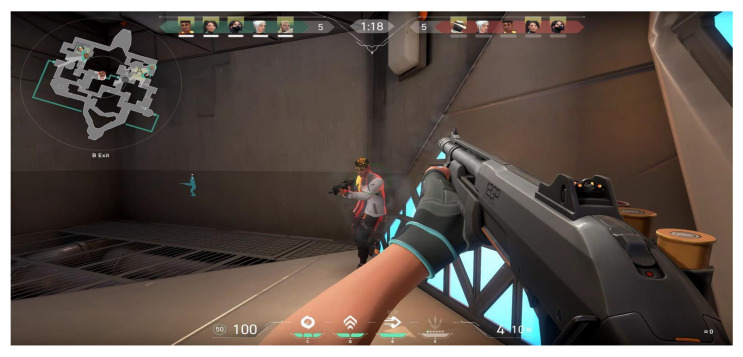
A duel in Valorant.

**Figure 5:**
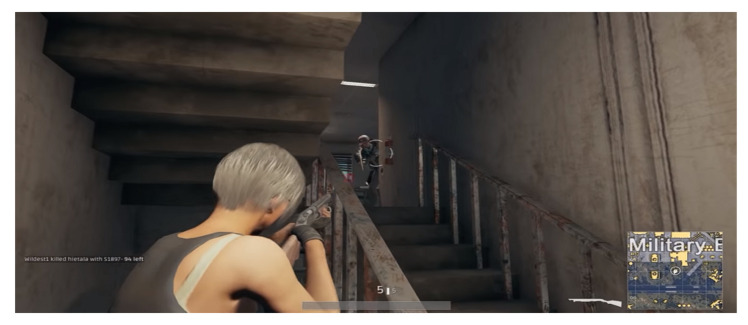
A duel in PUBG.

GunBlood, a traditional player versus player (PvP) shooter-type video game, is the simplest example of a shooter-type video game in terms of the parameters involved that can be examined using the theory of stochastic duel. Most of these games are played in a duel scenario, as seen in Figure 1. As previously discussed, more complex duel games include Counter Strike: Global Offensive (CS: GO) and Valorant, which require more strategic planning than a classic duel, since these involve teams of protagonists and antagonists but are limited to 10–14 players per game and have a relatively small area of roughly unit kilometer square in which the duels throughout the duration of the game take place. Figure 4 depicts the duels’ environment in these types of video games. Figure 4 shows a tiny space with no shelter and a clear line of sight. This simplifies the data provided by the game, making it easier to examine the duels that occur throughout the game. Video games such as PUBG and Free Fire properly reflect the most sophisticated version of a duel game, as the number of teams and the size of a team in these can be arbitrary. In addition to strategic planning, these games also require a good understanding of the player’s surrounding environment, stealth and team coordination, with an average of 98 players per game and an area of about 8 km containing different terrains, hence putting a lot more parameters in the view. Notice the dense cover available and the obstacles in the area adding to the available parameters in Figure 5. This implies that the data generated by the game in Figure 5 would have more parameters to analyze the duels that occur in the duration of the game. Figure 4 and Figure 5 give a visual comparison between the types of games for a better understanding of the duel environment different video games offer.

These video games generate enormous amounts of data, and since all of these games can be analyzed by the theory of stochastic duels, the data can be collected, which can help in proving the results of different models based on the theory. Based on the statistics presented by the data, the efficiency of these models may also be increased. The multiplayer feature of these new-age games is a plus, since, rather than being dependent on a machine, the data come from real players, resulting in a large variance in the values of many parameters provided by these games, resulting in better datasets for the models. These games’ online multiplayer gaming communities have a large number of members, and as a result, many tournaments and e-sports are organized each year. The data of these e-sports are often made publicly available, which makes it easy to access. The variance of data obtained in this fashion is also appreciable, since, as mentioned previously, all of these games are categorized as massively online multiplayer (MMO) in nature, that is, at any given point of time, players play against other live players instead of playing against the computer machine.

The rest of the paper is organized as follows. Section 2 of this paper contains brief history, relevant literature review and analysis of general interest in the subject. Further, Section 3 of this paper deals with results and demonstration of the proposed model. Then, Section 4 explains the implementation of the mathematical model as presented in Section 3. This is followed by Section 5, which outlines a few use cases and elaborates on the model’s future scope and civil applications. Section 6 brings the study to a close by highlighting the model’s assertions, summarizing the work conducted thus far and suggesting further research. Appendix A contains the link to GitHub repository where the implementation of the model has been hosted. Appendix B contains Table A1, which provides the list of acronyms used in the paper. 

## 2. Brief History and Literature Review

After the introduction of the theory, early works include the introduction of the fundamental duel game model and the classic duel game model. To solve the challenge of studying duels and combats, a variety of approaches were explored. Researchers attempted to divide the problem down into smaller, easier-to-understand portions in the early years of its debut. The fundamental duel between two players was broken down into the marksmen problem [3], which instead of directly analyzing the combat between two players, broke down the duel into a situation where both players shoot at different static targets.

This aided in obtaining the information needed to analyze the fundamental duel. The use of the theory was addressed in terms of many concepts and circumstances. During the 1980s, the concept of many duels as part of a larger battle was introduced. Following that, the frequency with which work on the idea was published dropped dramatically. Most of the work conducted before the 2000s revolved around the military aspects of the theory and, as the world progressed toward being more technologically driven, interest regarding the study of stochastic duels decreased over time. As famously quoted by Carl Von Clausewitz, “War is nothing but a duel on a larger scale” [15], and hence, if analyzed correctly, it could help avoid a lot of casualties. This concept of seeing a conflict between two forces as a duel on a larger scale reignited the interest in the theory of stochastic duels, as terrorism grew in the world in the first decade of the 2000s [7]. The potential for civil applications in the theory was only discovered considerably later in the second decade. Models were developed to address business- and market-related issues and to develop strategies in response [16,17]. A new viewpoint was developed, which saw n-player combat as a series of repetitive duels [18]. Most recently worked upon by Song-Kyoo (Amang) Kim, a hybrid model was introduced to try and solve various duel-type problems more effectively [18], talking about its use cases in building business strategies. Furthermore, the antagonistic one-to-n game model [17] discusses its application in market strategies. Although the modifications to the original model allow it to be theoretically employed for a variety of purposes, they do not account for the new model’s reliability and efficacy in real-world settings. The efficiency of these models can be accounted for by using the data generated from video games as mentioned in Section 1.3. The implementation of the model introduced in this paper mentioned in Appendix A uses the data from these shooter-type video games to support its results and hence shows an elaborate demonstration of the model.

Brief reviews of the mentioned works are provided below in order to gain a better understanding of stochastic duels and game theory, while also staying current with previous work. This allows us to assess the present state of development using the theory, as well as confirm our belief that the majority of the effort is focused on military sciences.

The model discussed in Ref. [7] shows a duel model between two forces. On the one hand are the guerrillas, and on the other are the organized forces. The guerrillas have the advantage of being able to choose the time and place of their engagement. The study demonstrates how to calculate the various advantages of a combat between organized forces and guerrillas. The article also reveals that the guerrilla side’s advantage grows when compared to the offensive side. This is because the latter has a better weapon system. The expressions of the winning probabilities of the duel are practical. Although the model is complex, the results are satisfying.

The antagonistic duel game model in Ref. [16], where the goal is to kill all the other players, was studied. It lets players experience different variations of the game at random. The time-dependent duel game model is derived by utilizing the first exceed theory after it is solved analytically. The concept of the model can be directly applied to real-world situations, especially those where the goal is to hit all the other players simultaneously. The analytical approach is then supported by the case studies related to the blue ocean market.

The recently published stochastic game model in Ref. [17] presents a model that has a continuum of states under a duel-type configuration. It offers an overview of the numerous tactics that may be utilized to acquire the best winning chance in a game and delivers a compact closed joint functional framework for strategic decisions in a game. The study investigates a novel sort of hostile duel game that permits participants to experience future iterations at random. A joint function of the standard stopping game was then constructed to analyze the best strategies of the players. The paper obtained compact closed forms from the Laplace–Carson transform. The hybrid game model was then presented as a solution for handling duel-type-game issues that was more flexible and effective. The paper also included an application for a smart-phone-based stochastic duel game.

An extended version of stochastic duel game is presented in Ref. [18] that make use of the antagonistic duel-type configuration. An analytical framework was developed to analyze the core of a stochastic duel game. After that, a robust method was utilized to create a time-dependent model that accounts for the various strategies of pair-wise players. Unlike typical dueling games, each battlefield is introduced initially, and each player chooses a target at various points of the game. The rules of a simple duel game are only allowed for pair-wise players in the field. The battlefield indicators also provide the most threatening player. The study of antagonistic games led to the development of new variants of the game that can be expanded to include multiple-player games.

An early attempt is undertaken by the author in Ref. [19] to regroup all the different results formulated regarding the theory of stochastic duels and declassify a lot of results that were previously inaccessible. The first section of this article includes the methodology used to construct the results, while the second section contains the actual results. It helps us obtain a sound understanding of the different approaches taken by authors throughout the world before the 1980s to derive their respective results.

General solutions for a stochastic duel with continuous interfering times have been discussed in Ref. [20], where the possibility of kill by multiple hits and fixed duel time are considered. The limitation of initial ammunition supply is also considered. To quantify the lethal impact of many strikes, two different methodologies are developed: “vital hit” and “damage coefficient.” The conclusions are supported by examples of negative exponential shooting times and geometric ammo supplies.

The works of Ancker in Ref. [2] have been extended by the author in Ref. [21] to account for the kills achieved by multiple hits. Previously worked upon results of the limited ammunition duel are discussed and are revised to include the “kill by multiple hits possibility.” Multiple single shot firing and burst firing modes are also discussed. A pattern of firing is used to explain the burst firing situation. The conclusions are supported by examples of negative exponential shooting time and geometric ammo supply.

A conceptual framework for stochastic duels is provided in Ref. [22], which develops a modest extension for realistic combat situations. It shows that the probability of winning a duel is derived by estimating the firing times at random times. The stochastic model’s classical and fundamental duel variations are investigated and assessed. Two scenarios are considered for the extension to multiple duels: (1) discrete firing times and (2) continuous firing times. Using the theory of continuous-time Markov chains, the chance of winning a duel is calculated by predicting the firing timings at random times.

A two-on-one scenario is discussed in Ref. [23], which builds upon the foundations of the original one-on-one stochastic duel [1] and derives the state equations, win probabilities, mean value and variance functions for a two-on-one scenario. The two-on-one stochastic duel is introduced for the first time, and a comparison is drawn between the case where one side has Erlang [2] firing times, and the other is negative exponential in the corresponding “stochastic Lanchester” and “Lanchester” models, hence proving their non-equivalence.

State probabilities for the previously introduced general many-on-one duel model have been derived in Ref. [24] based on the order in which targets are attacked. It provides evidence for its findings by portraying a three-on-one scenario. In a scenario where one side outnumbers the other, but the other is granted a firepower advantage, the relative efficacy of both sides is explored.

A practical application of the theory in a mountain battle scenario is discussed in Ref. [25]. This battle scenario is described in detail, and the role of terrain in the model is justified. The advantage of the defending side is elaborated and taken into account, while also keeping a check on the disadvantage of the attacking side, as they are forced to follow a narrow, winding and steep road. This scenario is treated as multiple many-on-one sub-duels, where multiple units of the defending side engage with the single exposed unit of the attacking side.

Many types of mathematical models have been developed to describe duels between two weapon systems. Prior to the development of new models for each tactical situation, duels were not explicitly defined. The general formulation of a model is discussed in Ref. [26] that shows the results of a duel between two weapons with varying firing times. By incorporating a variable firing time parameter, it intends to improve the study of duels. This improvement eliminates the discontinuities that were present in the previous model.

The use of game theory in economics is described in Ref. [27]. However, it does not use a duel or a combat model to come to grips with the challenges in the field of economics. It discusses how game-theoretic models help economists study the implications of rationality in the markets. It majorly focuses on elaborating the usage of game theory in the economic sector by leveling down the complexity of it and introduces some basic solution concepts and definitions.

An extension from the one-on-one stochastic duel model was drawn in Ref. [28] to the many-on-one case-based information sharing. In this many-on-one situation, a probability density function of the time it takes to kill a target is calculated. Using an example of exponential distributions of searching and firing times, the results are explained.

A two-person zero-sum game type model is discussed in Ref. [29] in a stochastic setting. The procedure of this game is described in the paper, and an interactive algorithm is given, which computes the optimal strategy. It also takes into account the situation where one of the armies has only one unit.

A modeling framework for evaluating fighter aircraft is presented in Ref. [30]. It can be used to evaluate the various components of an aircraft system due to the large capital investment involved in developing such systems. Its goal is to give a first-order analysis of a combat aircraft system in order to help decision makers make better judgments. A two-phase combat between two jet fighters is the subject of the model. These phases involve developing a Markov chain model to model the dogfight. In addition, the aircraft’s specifications are combined with various flight theories to provide a more accurate representation of the dynamics of the fight.

A review of the available literature on the use of game theory in defense applications is provided in Ref. [31]. It discusses previous papers on game theory in detail and classifies them based on different criteria. These criteria include one player, two player, multi-player and other criteria based on the command-and-control warfare.

The principles of game theory are applied in Ref. [32] to analyze conflicts between organizations and the public. A duel typesetting game is contrasted with a cooperative negotiating game between an organization and the general public. This is one of the first attempts to use game theory and a duel format to solve a civic matter. However, the duel game considered in this paper is not a stochastic process, but it does build a foundation to work upon in reference to civil problems.

A game-theoretic model is introduced in Ref. [33], which attempts to merge the principles of game theory and duel game model. The model is divided into two halves. There are two types of duel: (1) the noisy duel and (2) the silent duel. In the form of a continuous firing model, this concept is summarized and generalized. The noisy duel is described as one in which the strategy of one player is known to the other and is contrasted by the silent duel. The paper describes its usage in economic situations, such as bidding.

A brief review of some literature related to deep learning and its usage is conducted below, keeping in view the implementation of the model described in this paper. This is carried out in order to ease in the reader with some basic concepts of the deep-learning approach used in the implementation.

A review of recurrent neural networks is presented in Ref. [34]. It briefly elaborates on the Long Short-Term Memory model and discusses its use in the network architecture. A few variants of LSTM models are explored, and their applications are discussed. Some future research directions are also presented.

Some computational models are discussed in Ref. [35] that incorporate the use of deep learning to improve the state of the art in speech recognition, visual object recognition and other fields, which operate under the deep-learning umbrella.

The fundamentals of recurrent neural networks (RNN) are discussed in Ref. [36] to lay the foundation for LSTM models. It identifies new opportunities for the usage of LSTM models and produces the most general LSTM variant.

A real-life application of RNNs is discussed in Ref. [37], which explores the opportunity to use deep-learning models to improve the prediction model for stock price. The paper also examines suitability as prediction factors with various combinations of existing assistance indices through the R neural network package.

A brief outline of the developments made in the theory over the course of time as discussed above, relevant to the model introduced in this paper, is given in Table 1. This table gives us a timeline of published works and helps us understand and track the different ideas that were introduced in the field of the theory of stochastic duels.

The study of these variations is important to build a strong foundation for future work. A careful observation of Table 1 gives us an overview of how different types of models were used to cope with the real-life scenarios, along with the type of scenarios in terms of military or civil application domains.

**Table 1:** Variations introduced in the stochastic duel game model over time.

S. No.	Year	Resource	Novelty(s)	Intended Application Domain	Type of Proposed Model			
					One-on-One	Two-on-One	Many-on-One	Many-on-Many
1	1963	Stochastic Duels [2]	Theory of stochastic duels introduced.	Military	✔	✗	✗	✗
2	1966	The status of developments in the theory of stochastic duels—2 by Ancker Jr, C. J. [3]	The fundamental duel introduced, and developments in basic duel theory with respect to military combats discussed.	Military	✔	✗	✗	✗
3	2011	A kind of stochastic duel model for guerrilla war—Liwei Liu, Jun Yua, ZhiGuob [7]	Concept of war as a duel between teams (forces) introduced. Combat between terrorists and organized forces discussed.	Military	✗	✔	✗	✔
4	2020	Antagonistic One-To-N Stochastic Duel Game by Song-Kyoo (Amang) Kim [16]	“One player shooting to kill all others” concept introduced, and application in Red/Blue Ocean markets discussed.	Civil	✗	✗	✔	✗
5	2020	A Versatile Stochastic Duel Game by Song-Kyoo (Amang) Kim [17]	Time-based stochastic game model introduced, and application in business strategies discussed.	Civil	✔	✗	✗	✗
6	2021	Robust Pairwise n-Person Stochastic Duel Game by Song-Kyoo (Amang) Kim [18]	Concept of n-players in multiple battlefields introduced following a pair-wise duel.	Civil	✔	✗	✗	✗
7	1979	The One-on-One Stochastic Duel: Parts I and II—Anker Jr, C.J. [19]	A summary of all the formulations conducted to date is given to help future research.	Military	NA	NA	NA	NA
8	1980	Stochastic Duels with Multiple Hits, and Fixed Duel Time—Kwon, T.; Bai, D. [20]	Constraints on the original stochastic duel model applied. Results are discussed for duel with fixed time and are extended to multiple hit–kill possibilities.	Military	✔	✗	✗	✗
9	1983	Stochastic duels with multiple hits and limited ammunition supply—Kwon, T.; Bai, D. [21]	Constraints on the original stochastic duel model discussed. Results discussed for duel with limited ammunition supply, and possibility of multiple hits considered.	Military	✔	✗	✗	✗
10	1983	Some stochastic duel models of combat by Jum Soo Choe [22]	Results for multiple-duel model discussed for discrete and continuous firing times.	Military	✔	✗	✗	✗
11	1984	The two-on-one stochastic duel—Gafarian, A.; Ancker, C.J. [23]	A new model of two-on-one type is introduced, and results for the same are derived.	Military	✗	✔	✗	✗
12	1987	The many-on-one stochastic duel by Kress, M. [24]	State probabilities for the many-on-one model are derived, and results are illustrated using an example.	Military	✗	✗	✔	✗
13	1992	A many-on-many stochastic duel model for a mountain battle—Kress, M. [25]	Practical application of the theory of stochastic duels in a mountain battle scenario and its results are discussed.	Military	✗	✗	✗	✔
14	1993	Explicit modeling of detection within a stochastic duel—K. Wand, S. Humble, R. J. T. Wilson [26]	Concept of target detection in a duel introduced. Duel between two weapon systems discussed.	Military	✗	✔	✗	✗
15	1997	An Introduction to Applicable Game Theory—Gibbons, R. [27]	An economic use case of gametheory is explored, and usability is justified with an example.	Civil	NA	NA	NA	NA
16	2012	The Many-on-One Stochastic Duel Model with Information-Sharing—Li, J.; Liu, L. [28]	A many-on-one extension is drawn from the original one-on-one stochastic duel model, and results are derived on the basis of information sharing.	Military	✗	✗	✔	✗
17	2013	New results on a stochastic duel game with each force consisting of heterogeneous units by Kyle Y. Lin [29]	A two-person zero-sum game is discussed as part of combat between two forces, and an algorithm to compute the strategy is given.	Military	✗	✗	✗	✔
18	2017	Aircraft Evaluation Using Stochastic Duels—Gay, Jason W. [30]	Evaluation of performance of fighter aircraft in air combat conducted, and one-on-one aerial combat discussed.	Military	✔	✗	✗	✗
19	2022	Game Theory in Defence Applications: A Review—Ho, E.; Rajagopalan, A.; Skvortsov, A.; Arulampalam, S.; Piraveenan, M. [31]	Literature review of many attempts to use game theory in decision-making scenarios for military-based application is conducted. Details about specific models are also discussed.	Military	NA	NA	NA	NA

✔: Yes, ✗: No, NA: Not Applicable.

## 3. Proposed Model

In this proposed model of the game, unlike the n-person stochastic duel game [8], the only choice for a player once they encounter another is to fight. In addition to that, each player in the game identifies either as an antagonist/fraudulent player or as a protagonist/legitimate player. We distinguish between players based on their intentions, allowing us to improve the previously developed game model [9] to match more realistic requirements (use cases as discussed in Section 5). Each combatant in a duel is classified as either a killer or a victim. In addition to that, each player is also part of a team of an arbitrary size. All teammates validate each other’s trust, i.e., they support the actions of the player. Trust is defined for every player in the game on the basis of intent by a fraction with its value ranging from 0 to 1 with 0.5 as the threshold of neutrality (φ), which implies that if the player has their trust below 0.5, they are considered an antagonist, and if the player has their trust above 0.5, they are considered a protagonist. The mechanism of trust evaluation is further discussed in detail in Section 3.1.

The model can be setup in two different environments, namely, the antagonistic environment and the protagonist environment. The condition for ending the game is different for both of these environments. The game ends for the protagonist environment, and the protagonists are considered winners when 51% of the players reach a trust value above 0.5. This situation is considered to be the inflection point, where the majority of players in the game are protagonists/legitimate. The antagonistic environment is essentially an extension to the protagonist environment, where the game ends when the trust values of 51% of the players fall below 0.5 after rising above the threshold previously. This situation is considered to be the inflection point, where the majority of players in the game are antagonists/fraudulent. A simulation of the said model was carried out on a large publicly available dataset [24], as discussed further in Section 4, and future values of the time of reaching the end of the game and the median (0.5 quantile) of the trust values of all players were predicted (ref. Appendix A). Multiple application cases for this new model are discussed in Section 5, and the model’s flow is explained in Section 4 by Algorithm 1.
**Algorithm 1:** Simulation of model on the dataset
**Input:** Dataset of players, duels, time of duels.
**Output:** Time series of 0.5 quantiles, updated trust values of timesteps.1.**for** i in set of duels (Equation (3)) **do**2. evalTrust(timeSet[i], trustSet[i][victim], i)3.Initialize timeSeriesOfQuantiles array4.**for** i in trustSet **do**5. median ← trustSet[i].quantile(0.5)6. timeSeriesOfQuantiles.append(median)

To elaborate the model efficiently, let us consider the set of players:

(1) $$P=\left\{\;A_{1},\;A_{2},\;A_{3},\ldots ,\;A_{N}\;\right\}$$

where *A_i_* represents a player in the *N*-player game, and *N* is the total number of players. The different teams in the game are given by the set in Equation (2):

(2) $$Teams=\left\{\left(A_{a},\;A_{b},\;A_{c},\;A_{d}\right)\;\forall \;A_{a},\;A_{b},\;A_{c},\;A_{d}\;\epsilon \;P,\;a,\;b,\;c,\;d\;\epsilon \;\left[1,\;N\right]\right\}$$

The duels occurring during the game are given by the function in Equation (3):

(3) f: T→D

where

(4) $$T=\left\{\;T_{1},\;T_{2},\;T_{3},\ldots ,\;T_{N/2}\right\}$$

*T* in Equation (4) is the set of time steps, such that

$$T_{i}<=T_{j}\;\forall \;i,j\;\epsilon \;\left[1,\;N/2\right]$$

And

(5) $$D=\left\{\;{\left(a,b\right)}_{i}\;\forall \;a,\;b\;\epsilon \;P,\;i\;\epsilon \;\left[1,\;N/2\right]\;\right\}$$

Equation (5) is the set of duels between two players from the set of all players, such that the *i*-th duel (*a*, *b*)*_I_* occurs at *T_i_* time, with *a* being the killer and *b* being the victim. Let the set of trust values of players in the game be given by the function in Equation (6):

(6) θ: T→TrustSets

where

(7) $$TrustSets=\left\{\;Q_{i}:\;i\;\epsilon \;\left[0,\;T_{N}\right]\;\right\}$$

“*TrustSets*” in Equation (7) is the set of functions corresponding to *i*-th time step values from the set of time steps *T*, such that function *Q* in Equation (8)

(8) Q: P→K

where

(9) $$K=\left\{\;K_{i}:\;i\;\;\epsilon \;\left[1,\;N\right]\;\right\}$$

**Figure 6:**
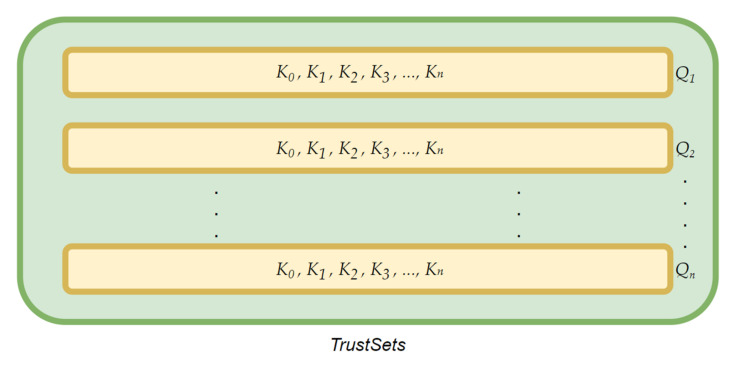
Representation of storage of trust data.

*K* in Equation (9) is the set of trust values of all players in the game, and *K_i_* is the trust value of *i*-th player from the set of all players, and *Q* is a function from the set of players to the set of trust values. Figure 6 explains the storage of trust data through pictorial representation. The green box in the picture represents the *TrustSets* as in Equation (7), which is essentially a set of multiple sets, all of which correspond to different time steps, and the yellow boxes show the set of trust values of players at any given time step. Further, the yellow box contains all the trust values for players at a particular time step. It is important to note that the size of the *TrustSets* as in Equation (7) would be equal to the number of time steps in the data used.

Each of the iterations on the set of duels (Equation (5)) represents a new timestep at which the duel takes place. On each of the iterations, trust is re-evaluated for the antagonist team involved in the duel based on the trust of the victim. At *T*_1_ = 0, each player is assigned a 0-trust value. On the first iteration, the first duel from the set of duels D occurs. Let us assume in this first duel, (*A_i_*, *A_j_*) are involved, where $i,j\;\epsilon \;\left[1,\;N\right]$, out of which *A_i_* shoots and kills *A_j_*. After the duel is over, *A_i_* turns out to be the killer in this duel.

### 3.1. Trust Evaluation

After every duel, the winner of the duel and their teammates’ trust are re-evaluated broadly based on three metrics:

- Honesty: Honesty is generally considered as a measure of integrity of a result/statement or a completed task by a player. In this instance, we use the player’s current trust value and compare it to the neutrality level to determine the player’s honesty.
- Lifespan: Lifespan is a strong metric to consider while evaluating the trust of a player, since it accounts for the current trust value of the player. A longer lifetime indicates that the present trust value is more trustworthy.
- Reputation: Another significant indicator is the player’s reputation among others, since it ensures that the player’s colleagues are also influenced by the duel because they support the player.

The above-mentioned metrics are nested under two different categories based on the impact they have on the trust of the player, which are, namely, direct trust and indirect trust.

- Direct Trust: Direct trust consists of the information directly obtained from the duel or the player. In this situation, we use the normalized value of the time step at which the duel takes place, i.e., the victim’s lifespan, as a direct measure for calculating trust. The normalization of time step is carried out by the following formula in Equation (10):

(10) $$T_{0}={\frac{T_{i}-T_{1}}{T_{N}-T_{1}}}_{}$$

where, *T*_0_ is the normalized value of *T_i_*, and *T*_1_ and *T_N_* refer to the time of occurrence of the first and last duels, respectively. Therefore, direct trust in Equation (11) is given by

(11) $$T_{DT}=T_{0}$$

- Indirect Trust: Indirect trust refers to the information obtained from the players involved in the duel. In this scenario, we use the victim’s trust value divergence from the neutrality threshold, i.e., the winner of the duel’s honesty, as an indirect metric for trust evaluation. Therefore, the indirect trust in Equation (12) is given by

(12) $$T_{IT}=0.5-K_{i}$$

In addition to the above-mentioned two metrics, the reputation of the killer is evaluated by updating the trust value of teammates of the killer along with the killer’s, since they support the killer.

Therefore, trust of *A_i_* is re-evaluated by following the below formula:

(13) $$T_{new}=sigmoid(T_{old}+\left(W_{DT}\times T_{DT}+W_{IT}\times T_{IT}\right))$$

where *T_new_* is the new trust of *A_i_*, *W_DT_* is the weight associated with direct trust, and *W_IT_* is the weight associated with indirect trust. This re-evaluation is carried out for all of the killer’s teammates in relation to their *T_old_*. To keep the trust levels in the 0 to 1 range, the sigmoid activation function is utilized. This process of trust re-evaluation is carried out on each of the iterations until the last duel occurs. Therefore, the whole process of trust evaluation can be described by Algorithms 2 and 3, as presented in Section 4. We assume both weights to be 0.5 in Algorithm 2.

Once all the iterations are completed, the trust values can be evaluated to find out the required time step as per the chosen environment.
**Algorithm 2:** Trust Evaluation
**Input:** Current timestep, trust value of victim, index of duel in set of duels (i)
**Output:** Updated trust values of players for given timestep1.**procedure** EVALTRUST(time, vicTrust, i)2. normTime ← normalizeTime(time)3. newTrustAdj ← $\left(0.5*\mathrm{normTime}+0.5*\left(0.5-\mathrm{vicTrust}\right)\right)$
4. **for** j in team of killer (Equation (2)) **do**5.  updatedTrust ← trustSet[i−1][j] + newTrustAdj6.  trustSet[i][j] ← sigmoid(updatedTrust)7. **for** k in set of players (Equation (1)) **do**8.  **if** k not in team of killer (Equation (2)) **then**9.   trustSet[i][k] ← trustSet[i−1][k]

**Algorithm 3:** Normalize Time
**Input:** Current time
**Output:** Normalized value of given time1.**procedure** NORMALIZETIME(currTime)2. minTime ← time of occurrence of first duel3. maxTime ← time of occurrence of last duel4. return($\left(currTime-minTime\right)/\left(maxTime-minTime\right)$)

### 3.2. Theoretical Analysis of the Model

The introduction of the trust evaluation system in the proposed mathematical model can be justified as follows.

**Hypothesis** **1** **(H1).** *The times of occurrence of duels are discrete.*

**Hypothesis** **2** **(H2).** *Trust values of players are fractional and belong to the interval (0, 1).*

**Hypothesis** **3** **(H3).** *The player with trust value 0.5 is neither an antagonist nor a protagonist.*

**Lemma** **1.** *Trust evaluation of players expands the model’s domain and makes it adaptive in nature.*

**Proof** **of** **Lemma** **1.** As mentioned in Section 3.1, the calculation of trust value of a player is carried out on multiple metrics to ensure a little variance in data, which is given in Equation (14) by

(14) $$variance=\sqrt{\frac{\Sigma {\left(x_{i}-\bar{x}\right)}^{2}}{n-1}}$$

where, *x_i_* is the value of one observation, $\bar{x}$ is the mean value of all observations, and *n* is the total number of observations. This variation also ensures that the result of the model simulation has a high usability score. Additionally, since more metrics can be added to the existing evaluation mechanism, it also ensures that the model has the ability to adapt to different types of data. Hence, trust evaluation expands the model’s domain. □

**Lemma** **2.** *The median (0.5 quantile) of trust values of all players at any given time step represents the least upper bound of the majority of players in the match.*

**Proof** **of** **Lemma** **2.** Median value separates a set into an upper half and a lower half and is given in Equation (15) by

(15) $$median=\frac{n}{2}th\;\mathrm{value}\;$$

From the beginning, when *n* is odd, and in Equation (16)

(16) $$median=\frac{\left(\frac{n}{2}th+\left(\frac{n}{2}+1\right)th\right)}{2}$$

when, *n* is even, where *n* is the total number of elements in the set, provided the elements are arranged in an ascending order. As a result, we can safely assume that the *median* (0.5 quantile) represents the greatest trust value among the majority of participants in the collection. □

**Theorem** **1.** *Trust evaluation mechanism in the model ensures increased applicability of the model in modern civil and technological domains.*

**Proof** **of** **Theorem** **1.** By Lemma 1, we can conclude that the introduction of a trust evaluation mechanism increases the credibility and also enhances the adaptability of the model, and by Lemma 2, it can be observed that the model takes into account the trust values of all players without any bias. This demonstrates that the model’s trust mechanism secures and permits its usage in a variety of current civic and technological sectors. □

**Theorem** **2.** *The ability of the model to generate trust values without compromising variance in data enhances the accuracy in predictions made by the model, and hence, performs a better combat analysis.*

**Proof** **of** **Theorem** **2.** Lemma 1 accounts for the variance in data generated by the model. These data are generated in such a way that they are easy to feed to a deep-learning model further discussed in Section 4, and not much reshaping is required. A higher variance also improves the model’s accuracy because, unlike in this model, if the trust values thus generated had a very low variance, the sequence obtained would have been extremely difficult to predict accurately because any existing machine- or deep-learning model applied to such data would have produced repetitive values. If the results are predicted with good accuracy, it implies that our model is good at its job in performing an analysis of the combat/duels based on the trust values of players. Hence, it is safe to conclude that the good variance in generated values ensures good accuracy, which in turn causes the model to be better at combat analysis. □

## 4. Implementation of the Proposed Model

The mathematical model can be implemented, and the results for the same can be easily verified. To begin with the implementation, a dataset of duels and players is a prerequisite. Trust values of the players can be generated by simulating the model. For the ease of understanding, the simulation process can be divided into two modules: (1) the main simulation and (2) the trust evaluation. The algorithms used in the implementation of the model proposed are given below. These are written in a standard pseudo form to help the understanding of readers.

At the start of the simulation, we create a trust values data frame, with each row indicating a time step and each column representing a participant. In a Python environment, the data frame containing the relevant data is loaded. We use a publicly available dataset comprising data collected by PUBG [38] for modeling purposes. From this data frame, we iterate over the set of duels using an “iterator” as described in Algorithm 1.Each of the iterations involves only one duel, and these iterations are carried out after sorting the set of duels based on the time step at which they occur, that is, the order of time step at which each duel occurs is strictly increasing.

During each of the iterations, the previously initialized trust values are re-evaluated. This trust re-evaluation process is described in Algorithm 2 presented in Section 4, which uses Equation (13) to calculate the trust values of the involved parties in the duel. The next step is to update the recalculated values, which involves appending the new trust values of all impacted players to an array of trust values corresponding to the current time step (iteration), as well as the prior trust values of unaffected players. At the end of each of the iterations, this array is merged into the trust data frame as a row.

**Figure 7:**
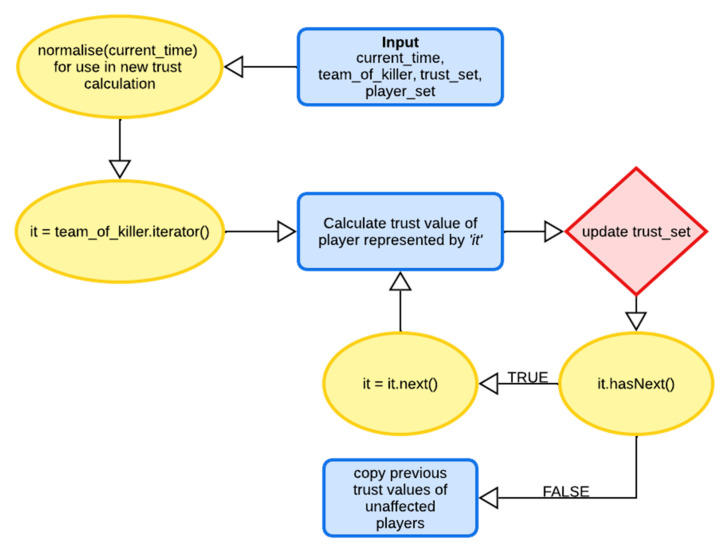
Flow of the trust evaluation module.

**Figure 8:**
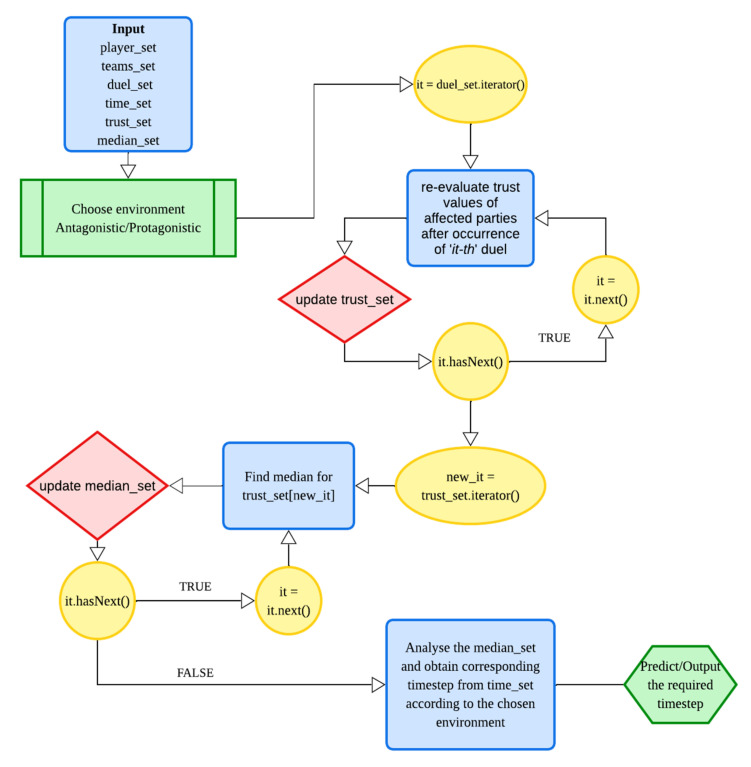
Flow of the main simulation.

**Figure 9:**
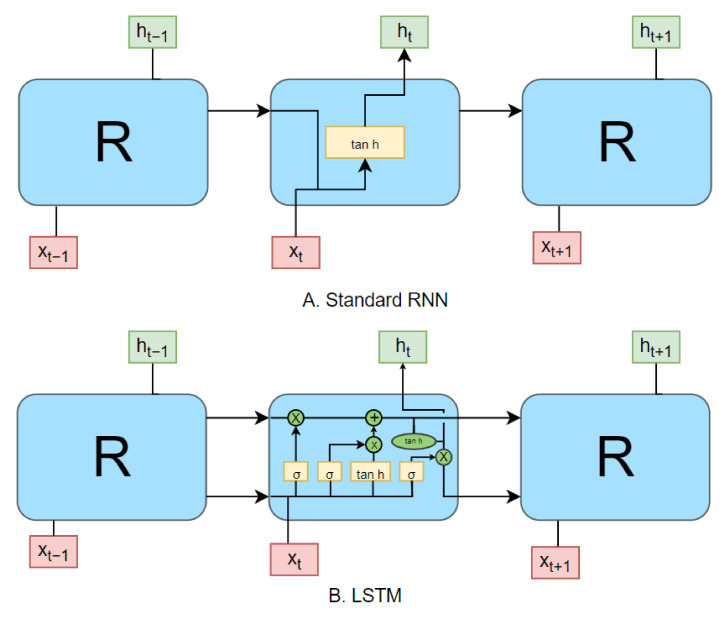
A standard RNN with single layer (**A**) versus LSTM with four layers (**B**).

The flow diagrams given in Figure 7 and Figure 8 represent the flow of the simulation and flow of the trust re-evaluation processes, respectively. Furthermore, predictions are done using deep learning based on the requirement of the chosen environment. The flow of data within the deep learning model can be seen in Figure 9.

In order to identify whether the end goal was reached in the duration of the game, we create histograms for all the elements of each row in the trust values data frame or *TrustSets* as mentioned in Equation (7). The 0.5 quantiles are derived from these histograms, generating a time series of 0.5 quantiles, which are stored in a set in an ordered fashion. The model may be classified into two settings based on this time series: antagonistic-type environment and protagonist-type environment.

These are essentially the two end conditions, which, when met, would result in the end of the simulation of the model. This would come in handy later to identify whether the goal had been achieved, as per the chosen environment.

In the protagonist-type environment, we find the time step when the 0.5 quantile value rises above the threshold value, i.e., when the protagonists’ dominance is established. Meanwhile, in the antagonist-type setup, we find the time step when the pre-existing dominance of the protagonists is broken and the 0.5 quantile falls below the threshold. If no such time steps are found in the duration of the game, future values of the time series can be predicted to suit the use case.

The prediction can be carried out with the help of deep learning [39]. Multiple approaches exist that make use of concepts of deep learning to predict the next element based on the sequence of previous elements [34,35].

In recent years, some major work has been conducted on Long Short-Term Memory (LSTM) models [37], which are best described as a variation of recurrent neural networks (RNN) [40,41]. Although in theory, the classic “vanilla” RNNs can do a good job in predicting forward sequences, in practice, training RNNs on large datasets leads to the exploding gradient problem or the long-term dependency problem. It essentially means that while training RNNs using the back propagation, the long-term gradients tend to become zero or even tend to infinity, often referred to as exploding, resulting in the “exploding gradient problem”. This problem has been dealt with in recent past.

Many different variations of the “vanilla” RNN have been published, which have their own flaws and merits. By analyzing and observing the dataset to be used in the implementation of the model introduced in this paper, we find the most recently worked upon LSTM model [40] to be a great fit to serve our purpose. Therefore, the next time step prediction can be performed using a LSTM model, which, as described above, is an artificial RNN architecture used in the field of deep learning. Additionally, because time series and quantiles are fundamentally sequences, it is an excellent option for this purpose because it comprises feedback connections rather than normal feed-forward neural networks.

Figure 9 shows the difference between the repeating modules in the standard RNN and LSTM. The yellow boxes represent neural network layers, while the green ovals represent point-wise operations. The arrows in Figure 9 are used to depict vector transfer. Values “x” and “h” are the received input and generated output, respectively.

It is important to note that the dataset being used for simulation of this model comprises multiple matches of PUBG. Therefore, the accuracy of predictions performed in the implementation (Appendix A) by using LSTM deep-learning models varies for different matches. However, the models are able to produce an average accuracy of around 92–95%. This justifies the usage of LSTM models for predicting the next time step in the time series and the next 0.5 quantile in the quantile series.

**Figure 10:**
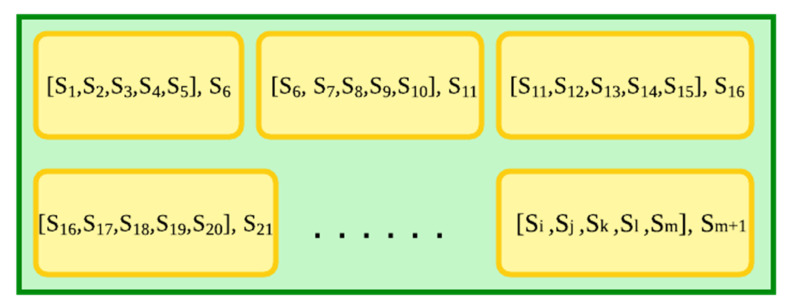
Structure of data frame fed to LSTM model in the implementation.

After obtaining the time series of 0.5 quantiles in the form of an ordered set, we break down the full sequence hence obtained into smaller subsets of constant size. This constant size plays an important role in predicting future values, as a data frame of these subsets is fed into the LSTM model, which is trained based on these data. This data frame is essentially an array of tuples where the tuples contain a subset of the time series of 0.5 quantiles and the next number in the sequence. Figure 10 shows a pictorial representation of the structure of this data frame, where S_i_ represents an element of the time series of 0.5 quantiles.

Once the model is trained, we can run it to predict the next 0.5 quantile in the time series. We refer to this model as the quantile LSTM model further in the paper for ease of understanding. A similar approach can be taken to predict the next time step by splitting the set of time steps into subsets and putting together another data frame, according to Figure 10, where Si would represent an element from the set of time steps.

The data frame is represented by the green box in Figure 10, and the smaller yellow boxes are the data frame’s constituents. Each element is a tuple, in essence. The array composed of the elements of the sequence is the first element of this tuple, and the second element is the next member from the sequence according to the array.

This data frame can be used in training another LSTM model, which would be able to predict the time of occurrence of the next duel. Let this model be called time step LSTM model. Once this model is trained, both of the models, the quantile LSTM and the time step LSTM can be run simultaneously on the provided data to predict both the time step and the median trust at that time step, as per the chosen environment of the simulation.

**Figure 11:**
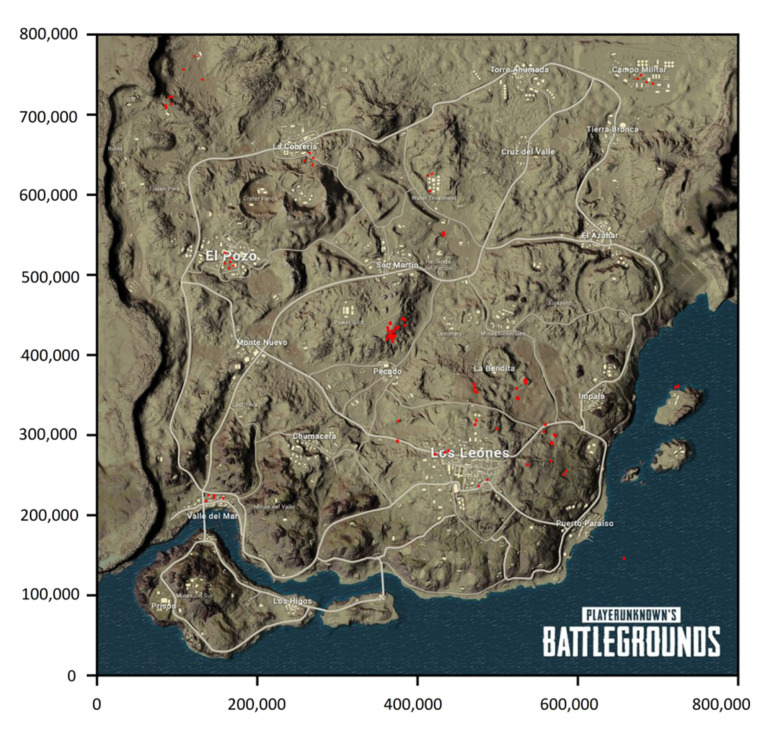
Duels occurring across the map throughout the game.

**Figure 12:**
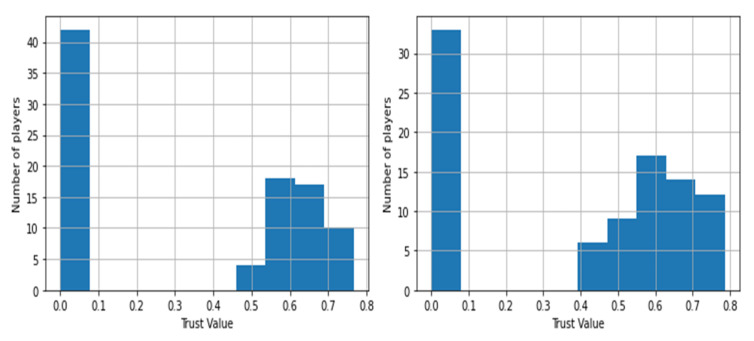
Histograms for 73rd and 74th time step from the implementation.

Figure 11 depicts the duels that take place throughout the game and are dispersed over the whole area. A duel is represented by each red marking on the map, which occurs at distinct time steps. Figure 12 gives an example of the histograms constructed from the trust values of the players in the game. The data used were uploaded along with the source code (Appendix A).

**Figure 13:**
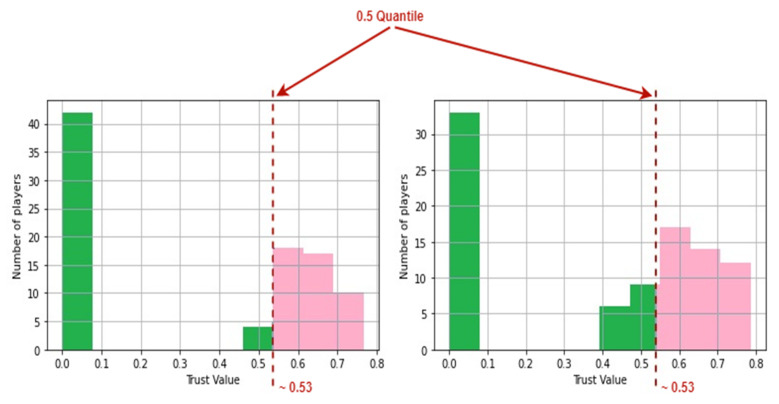
Observations from the histograms from the implementation.

**Figure 14:**
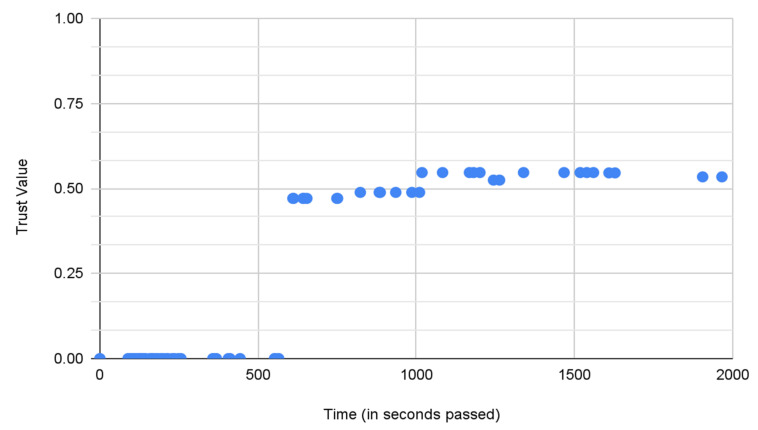
Scatter plot of the time series of 0.5 quantiles from the implementation.

Figure 13 shows the 0.5 quantiles obtained from the histograms. These 0.5 quantiles are the points in Figure 13 that divide the area covered by histograms into two equal portions and are depicted by the dashed red lines, implying that the area covered by the green and pink hues is equal. Therefore the pink and green portion of the plot signifies that the 0.5 quantiles bisect the area under graph. After obtaining the 0.5 quantiles from the histograms, Figure 14 displays the times series of the medians produced. This time series can help us find out the resulting median trust value and the time step at which any of the end conditions are met.

However, this may not be the case every time and is only applicable to cases where running the prediction model, as discussed above, is not required.

### 4.1. Model Review and Comparison

Previously proposed models so far [16,18] all relied on some specific parameters. For instance, the most recently worked upon models described in “Antagonistic One-To-N Stochastic Duel Game” [16] and “Robust Pairwise n-Person Stochastic Duel Game” [18] both rely on the availability of success probabilities of the players, which cannot be accurately determined in most practical cases, which reduces the applicability of these models in realistic scenarios.

The addition of a trust evaluation mechanism and the capacity of the model presented in this research to function on fairly realistic data contribute to its uniqueness.

**Figure 15:**
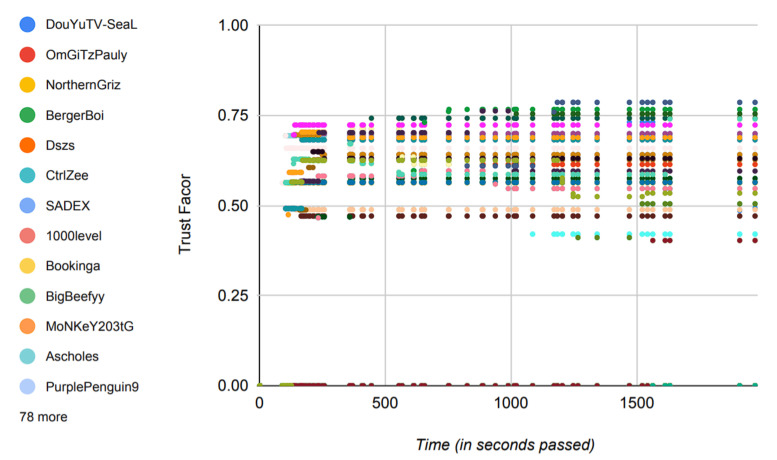
Variations in trust factors of players with time from the implementation.

Trust in itself is a very dynamic metric, which can consist of many different factors, a few of which are used in this paper and are discussed in Section 3.2. The model includes a detailed description to assist the user in determining the trust values. This allows the model to run on existing, actual historical data, making it a better and more reliable option for practical applications. Figure 15 justifies this notion by showing the variance in the trust values of players with time.

**Figure 16:**
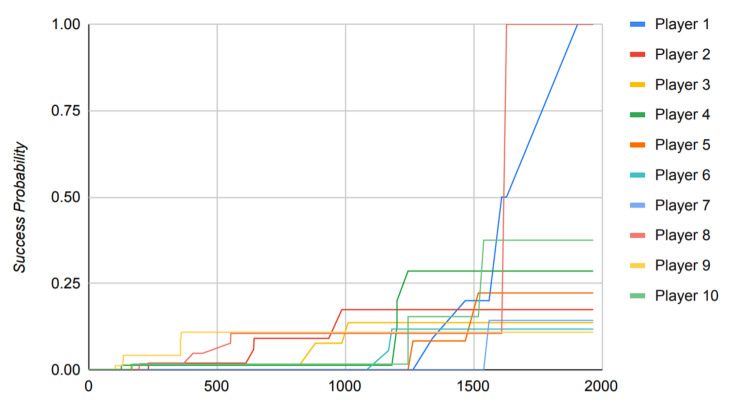
Success probabilities of top ~11% of players from a set of 91 players from a match of PUBG.

**Figure 17:**
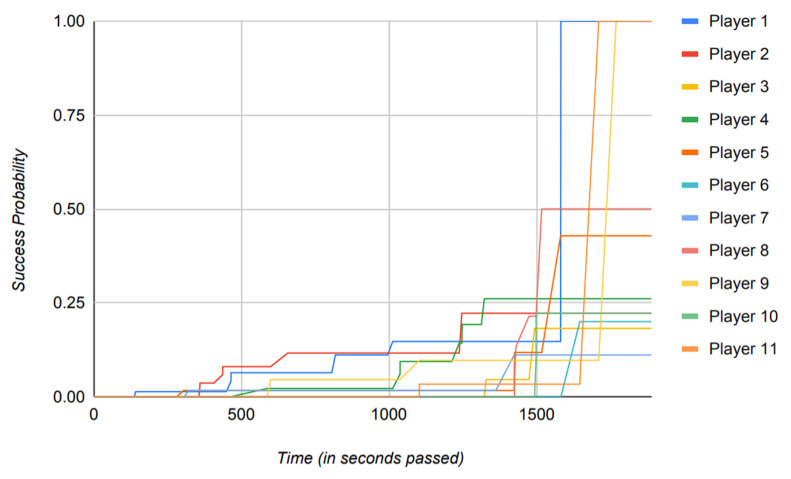
Success probabilities of top ~11% of players from a set of 99 players from a match of PUBG.

**Figure 18:**
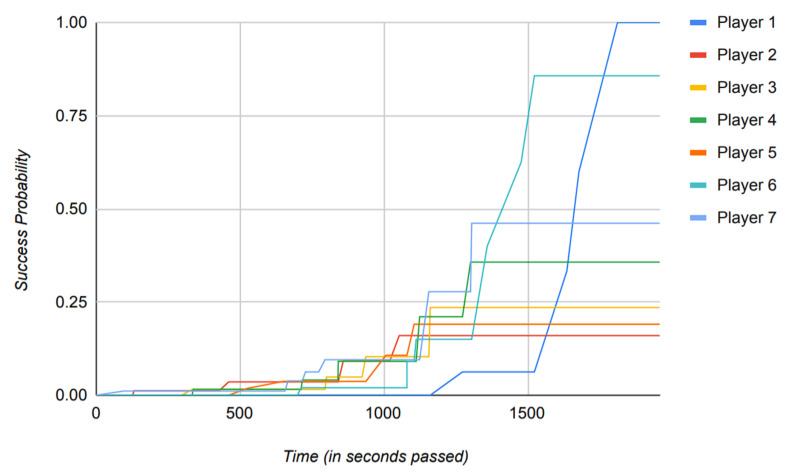
Success probabilities of top ~11% of players from a set of 82 players from a match of PUBG.

**Figure 19:**
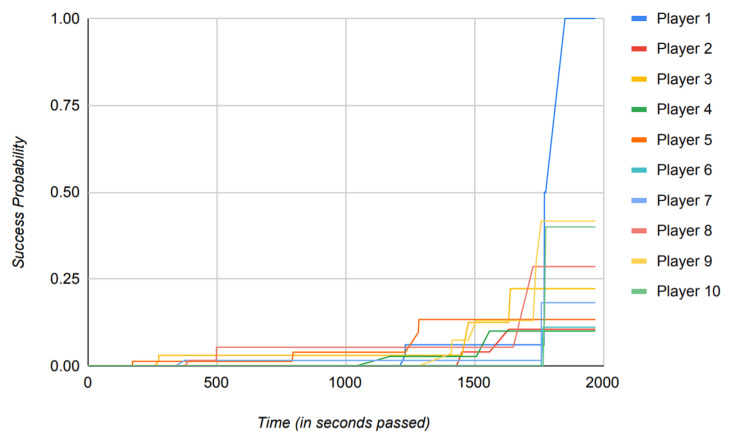
Success probabilities of top ~11% of players from a set of 90 players from a match of PUBG.

The reliance of the previously proposed models on success probabilities of the players in the game, as mentioned previously, proves to be of great harm when the model is put to use in a real-world scenario. Figure 16, Figure 17, Figure 18 and Figure 19 depict the outcome of the model provided in Ref. [18] when fed with a clean version of the data generated by different PUBG matches that were utilized in the evaluation of the model suggested in this paper.

Observing Figure 16, Figure 17, Figure 18 and Figure 19, it can clearly be seen that the success probabilities of the players in the real world differ quite a lot from the expected theoretical values as described in Ref. [18]. This finding supports our notion that success probabilities are appropriate when the data to be analyzed are modest or artificial/theoretical in nature. This was the case as described in Ref. [18], where the author designed a simulated game as part of the model. Table 2 presents a brief comparison of the model proposed in this paper with the previously published models. It highlights the key points, which add to the novelty of the proposed model and also count toward its strength. Furthermore, as previously noted, none of the recently published papers describe a precise approach for calculating the players’ success probability. This is a concern, since determining the probability of any occurrence properly is a difficult task in itself.

Additionally, to base the results of the whole model on such a metric is not practical and constrains the usage of the model to theory, hence restricting its usage in a real-world scenario.

Instead of using success probabilities of the players, the model in this paper works by generating the trust values of players from the already available data in the real world and then operating on those data to generate the results. It is important to note that the data shown in Figure 15 directly reflect the achievement of the proposed model to produce distinct trust values based on players’ attributes and, hence, depicts the reliability of the model if put to practical use.

This enhances the usability of the model in realistic scenarios rather than simply operating in a theoretical environment. In addition to that, the implementation of the proposed model is built using the data from a MMO game as opposed to Ref. [18], where the author works with the small game in a simulated environment.

From Table 2, it can clearly be observed that the model proposed in this paper defines a new standard of modern usage for the future models. Even though the software implementations of these previously published models, as described in Table 2, are open source, they do not provide an algorithmic approach in their published literature and work strictly on calculations of probability.

This makes the implementations of these models tougher to reproduce and amend, which in turn also halts the ability of these models to work on real-world data. It is also worth mentioning that, while previously released models detail their use cases in certain domains, they do not account for the model’s efficiency when applied to real-world data. This can clearly be observed from the above figures as well, where the actual outcomes of the success probabilities differ very much from the expected values as described in Ref. [17].

The results of Ref. [17] propose the success probabilities of all the players to reach 1 by the end of the simulated game. However, in a more realistic scenario, such as the one described in Section 4 where we use data generated by an MMO game such as PUBG to train the model, this is exceedingly improbable.

Another key point to be noted is that when the previously published models mentioned in Table 2 run against the data where the time scale is relatively large, they tend to perform poorly, which can clearly be observed from Figure 16, Figure 17, Figure 18 and Figure 19. Therefore, in conclusion, Figure 16, Figure 17, Figure 18 and Figure 19 contribute to our notion that the recently published models [16,18], however good theoretically, are unable to produce practically appropriate results and, hence, limit their use to theoretical discussions.

**Figure 20:**
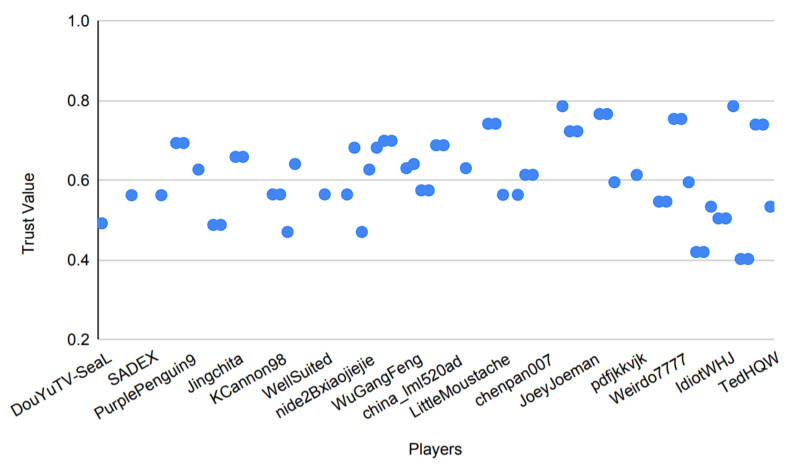
Maximum trust values of top ~20% of players achieved at the end of the match from the implementation.

Figure 20 shows the maximum trust values achieved by the final ~20% of players remaining in the game. This helps us identify the values, which are above the 0.5 quantile of the final timestep, as shown in Figure 14. This further strengthens our notion that the prescribed algorithms, as used in the model, are able to generate trust values with a good amount of variation and, therefore, are practically appropriate, as previously discussed in Section 3.2.

In contrast, our suggested mathematical model approach can run on enormous datasets, as seen by its implementation in Section 4, and thus provides a scalable solution. The teaming of players is another point that went unnoticed by the models mentioned in Table 2, since it is not strictly mentioned in any of the literature. By incorporating the idea of teams into the game, our suggested model addresses this issue. Other models do not allow one player’s actions to have a direct impact on another. However, this is generally not a good assumption to make, as most of the modern use cases include the clustering of nodes, which would pose a problem for these other models.

### 4.2. Innovation and Significance

The model displays its merits when compared to other recent models in Table 2. It can clearly be deduced from Table 2 that the model introduced in this paper improves the overall state of the art in the stochastic duel games field. It also puts forward some major courses of action and a new perspective for future research, as well as practical applications (as described in Section 6). The model is the first among others in the field of combat analysis and stochastic duel game assessment to incorporate deep learning in order to account for the efficiency of its results. It also presents a novel approach, which deals with the trust of the players. This approach contributes to solving modern problems through the use of stochastic duels and game theory. The new perspective of verifying the credibility of results through the use of datasets generated by shooter-type video games and the introduction of trust to extend the use of the theory of stochastic duels to modern problems are the main points of innovation in the introduced model.

### 4.3. Pros and Cons of the Proposed Model

As previously mentioned, the above-discussed approach presents a new perspective and lays down the course of action to enable the use of the theory of stochastic duels in solving problems relevant to the modern day. The approach makes use of the technological advancements that have occurred over the past to improve state-of-the-art models in the field. We use the datasets generated by video games to train and test deep-learning implementation of the mathematical model as described in Section 3. This helps us account for the efficiency of the model when put to actual use.

It is important to note that the incorporation of deep learning in the model comes with its own set of drawbacks. However, a good amount of effort was put into making the model as accurate as possible, keeping up with the available dataset and the actual requirements. Even though most of the use cases, as described in Section 5, offer great opportunities for the working of the model, many of them require the model to be run on a real-time basis. This, however minor an issue, can be seen as a possible problem that may hinder the adoption of the model in the real world.

Another issue that may arise during further research or implementation in use cases is the availability of data to be used by the model. To deal with this issue, the model provides proper methods to generate the data for essential parameters, such as trust and quantiles (as discussed in Section 4). However, it might not be feasible in certain cases to produce or gather the required data, which, again, is a problem faced by many deep-learning models.

## 5. Use Cases and Scope

A few practical use cases of the model proposed in this paper are given below. These may be worked upon in depth in the future.

- Blockchain

With the recent growth in the blockchain territory and the rise of decentralized finance (DeFi) and cryptocurrencies, the occurrences of 51% attacks on different blockchains have seen a dramatic increase in recent years. In addition to that, the relevance of blockchains has been on a significant rise in the recent decade, with many solutions tomodern problems being based on the blockchain technology [42,43]. Smaller blockchains with a low hashrate are more vulnerable to such attacks, as a lower hashrate means less processing power is needed to validate and add transactions to the blockchain. The model presented in this study may be used to maintain track of all the different nodes involved in block mining.

For instance, let us consider a situation where each node acting as a miner in a blockchain is assigned a trust value. This is analogous to the trust values assigned to the players, as described in the model introduced in this paper. Different mining pools may be used to identify clusters of nodes, which can then be deemed a team, according to the model. In this case, the process of mining a block would be equivalent to a duel game in the model if it were performed in real time. Let A be the winner node of the duel game among multiple nodes A, B, C,…,Z who try to mine the block, i.e., the block reward is given to the winner node (A). The victim’s trust in this situation can be taken as the median trust value of all other nodes, excluding one to which the reward for mining the current block was given, i.e., BtoZ, and then, trust evaluation for the next transaction can take place. If the model is run in a similar manner over time, the model will ultimately be able to determine the intent of the nodes using the trust values of the nodes, and if a node or a cluster of nodes begins to dominate the network of nodes, its trust value will reflect the same. If the model is pre-trained on historic data, predictions can be made based on these data to see whether it is possible for the median trust value of nodes to go below a certain threshold of neutrality. Due to the model’s flexible nature, the thresholds may be configured to examine the activity of different nodes and verify their purpose, which will help forecast any potential 51 percent attacks. Running this trust-based model, along with the proof of work mechanism, would result in enhanced security for the blockchain.

- Wireless Sensor Networks

Recent valuation of the wireless sensor network markets has predicted it to reach USD 148.67 billion by 2026, exhibiting a CAGR of 18.3% during the forecast period of 2019 to 2026 [44]. With such an imminent increase in growth rate, the probability of different attacks is greatly increased.

Impersonation attacks, such as a Sybil attack or a HELLO flood attack, may be foreseen and averted if the model proposed in this article is used correctly, since it provides a mechanism to determine the legitimacy of participants, who in this instance correspond to the nodes. The trust mechanism introduced in this paper can be applied in a real-time fashion to keep track of and generate trust values for nodes. These trust values can then be monitored on a real-time basis to keep a check on the intent of different nodes in the wireless sensor network [45,46].

The 0.5 quantile of trust values generated at any given period, assuming the model was previously configured to run for a long time, would yield a strong result reflecting the player’s purpose. This can be put to use by limiting the privileges enjoyed by the nodes that have their trust on the lower side of the set threshold. The sender and the receiver of packets may be compared to the killer and the victim in the model, and a transaction between the sender and the receiver is analogous to a duel game and can be compared to a battle in the model. The next element in the time series of 0.5 quantiles of trust values can be predicted by using the approach described in Section 4, which would indicate the strength of the network against impersonation attacks.

- Internet of Things

The global Internet of Things (IoT) market size was USD 308.97 billion in 2020. The market is projected to grow from USD 381.30 billion in 2021 to USD 1854.76 billion in 2028 at a CAGR of 25.4% in the 2021–2028 period [47]. It goes without saying that with this projected expansion, the number of IoT devices and users would skyrocket. It is undeniable that cyber-criminal organizations have used botnets made up of an enormous number of IoT devices to carry out various sorts of assaults in the past. The cybercriminals usually build a botnet by installing some kind of malware on these IoT devices, usually a Trojan, to carry out distributed denial of service (DDoS) attacks and send spam. Information stealth is another major issue when it comes to IoT devices. Both of these concerns may be addressed if the approach described in this work is used correctly. Besides the number of security principles, a number of attacks and their countermeasures at different IoT layers are available [48,49]. Impersonation attacks must be avoided at all costs in order to prevent data theft from IoT devices. This may be addressed by employing an approach similar to that used by WSNs, in which we run the model in real time to determine the validity of a connection attempt from a client to the IoT device. In this case, the connection attempt would be analogous to a duel game and could be compared to a duel in the model, and failed connection requests would correspond to the connecting device being considered the killer. The victim’s trust value may be calculated using the median of all presently connected node trust values, which will aid in computing the new trust of the connecting device that failed to authenticate successfully when the connection request was served [50,51].

“The botnet problem” can be avoided if only one source is permitted to install new software or update the current software. However, this is not a practical option, since in many cases, users must manually install updates for their own convenience and in the event that the installed software or downloaded updates become corrupted. Therefore, this model can be put to use to keep in check the intent of the installer or the origin of the software or update. In case the update is sent over the air, like in most modern cases, the intent of the origin server can be kept in check. This would work similarly to the processes of prevention of information theft using this model. Each installation attempt may be viewed as a duel game, with the victim’s trust being determined by the median of trust values from previously installed upgrades. This would aid in determining the trust of the owner of the update/software that is now being installed. It can be concluded from the above-mentioned analogies that a real-time version of this model with a few modifications would prove to be useful in preventing information theft and stalling the generation of botnets if implemented carefully.

In addition to employing the trust calculation approach put forward by the model, deep-learning implementation approach can also be put to use in order to achieve the goal of the paper. The fulfillment of the end conditions of the model under two environments, as described in Section 3, can be checked when the deep-learning implementation is performed after generating the required data by running the model on a real-time basis as previously discussed.

- Internet of Vehicles

According to one report [52], Internet of Vehicles (IoV) market size is projected to reach USD 369.60 billion in 2028 at a CAGR of 21.3%. In 2020, the Internet of Vehicles market was valued at USD 85.12 billion. Attacks on IoV are comparable to those on WSNs and IoT [53]. Various authentication attacks, such as the Sybil attack and wormhole attacks, can be prevented with a higher likelihood if the model presented in this work is implemented correctly and in real time. Similarly, to the previously described IoT scenario, each authentication request can be treated as a duel with respect to the model. The victim’s trust may be determined using the median value of all presently connected nodes’ trusts, and the authentication requestor’s trust can be recalculated using this value.

Availability attacks, such as denial of service and channel interference, can also be kept in check using the trust mechanism described in Section 3.1. A trust value can be assigned against the origin of all connection requests, which would help identify the intent of each requestor. The median trust value of all presently connected nodes can be used to recalculate the trust of each requestor. This technique, if carried out in real time, would aid in keeping track of all requestors, as the transactions are essentially analogous to duel games. Further, if an imminent attack is to be predicted, a deep-learning model, as described in Section 4, can be implemented using the data previously generated by running the model on a real-time basis.

Secrecy attacks, which try to eavesdrop or meddle in data transmission to acquire sensitive information, can also be dealt with in a similar manner. This may be addressed by maintaining a trust database for all nodes and therefore identifying lawful nodes to which sensitive data will be disclosed. In this case, likewise, the requests from the nodes can be treated as duels, and trust of the requestor can be re-evaluated based on the median trust value of all currently connected nodes. When correctly implemented, as per the needs and the provided manner, the model may clearly be seen to be fairly beneficial in preventing and predicting different listed threats.

In addition to using the model’s trust calculation technique, a deep-learning implementation strategy may be used to meet the paper’s aim. When the deep-learning implementation is finished after producing the requisite data by running the model in real-time, as previously stated, the model’s end conditions under two environments, as specified in Section 3, may be tested.

## 6. Conclusions and Future Work

In this paper, a conceptual foundation of the theory of stochastic duel was provided. Other methods of combat analysis and evaluation were discussed, and the dynamic nature of the theory of stochastic duels was justified. The theory’s use in video games was explored, as was the industry’s growth. The use of the data generated by shooter-type video games in verifying the results of the model and improving its efficiency was justified. Multiple stochastic duels models were investigated, and a novel model was introduced. The concept of trust evaluation was adopted to fit the model’s requirements. The proposed model also took into account the formation of teams of players. This new proposed model gives a new perspective to the original stochastic duels concept and also introduces a new approach for solving problems in the new world. Deep-learning models were created, and publicly accessible datasets were utilized to forecast the inflection points of several games (ref. Appendix A) based on their surroundings. The source code for the implementation of the model and the data sources are publicly available. Readers who wish to try out this new model for further research purposes may follow the GitHub link provided in Appendix A.

The model presented in this study is highly dynamic, and it serves as a foundation for future models that may be constructed upon it. Future work would therefore include models that are more specific and constrained to certain use cases, some of which are also discussed in the paper. When working on specific use cases, the trust evaluation method specified in the model can be changed based on the available data. The approach of using datasets of video games to verify the results and perform simulations of models would also come in handy when working upon the future models based on stochastic duel theory.

## Figures and Tables

**Table 2:** Comparison of the proposed model with previously published models.

S. No.	Feature	Antagonistic One-to-N Stochastic Duel Game [16]	A Versatile Stochastic Duel Game [17]	Robust Pairwise n-Person Stochastic Duel Game [18]	Proposed Model
1.	Software implementation of the proposed mathematical model	✔	✔	✔	✔
2.	Applicable modern use cases	✔	✔	✗	✔
3.	“Trust value” of players as a metric in calculation of results	✗	✗	✗	✔
4.	Methods for generating data for trust or other metrics to be used in calculation of results given	✗	✗	✗	✔
5.	Algorithms to justify the implementation	✗	✗	✗	✔
6.	Massively multiplayer online (MMO) video games to achieve better efficiency on results of the model	✗	✗	✗	✔
7.	Deep-learning approach in support of the mathematical model	✗	✗	✗	✔
8.	Ability of the model to work on an infinite time scale	✗	✗	✗	✔
9.	Ability of the model to work in a team-based environment where actions of the player affect their team	✗	✗	✗	✔

✔: Yes, ✗: No, NA: Not Applicable.

## Data Availability

There are no available data to be stated.

## Appendix A

The corresponding Python codes are available at GitHub (https://github.com/akarshroot/npcetm) (accessed on 19 February 2022) for the readers to try the model refer for the future research).

## Appendix B

**Table A1:** List of acronyms and symbols used in the paper.

Acronym/Symbol	Definition
BDA	Battle Damage Assessment
PvP	Player Versus Player
RPG	Role Playing Game
CS:GO	Counter Strike: Global Offensive
PUBG	Player Unkown’s Battlegrounds
USD	United States Dollar
LSTM	Long-Short-Term Memory
CAGR	Compound Annual Growth Rate
DeFi	Decentralized Finance
Φ	Threshold of Neutrality
Θ	Function of Trust Sets
F	Function of Duel
Ki	Trust Value of a Player
∀	For All
ϵ	Belongs to (Epsilon)
MMO	Massively Multiplayer Online Game
